# cAMP/PKA signaling balances respiratory activity with mitochondria dependent apoptosis via transcriptional regulation

**DOI:** 10.1186/1471-2121-11-92

**Published:** 2010-11-25

**Authors:** Jane E Leadsham, Campbell W Gourlay

**Affiliations:** 1 Department of Biosciences, University of Kent, Canterbury Kent, CT2 7NJ, England, UK

## Abstract

**Background:**

Appropriate control of mitochondrial function, morphology and biogenesis are crucial determinants of the general health of eukaryotic cells. It is therefore imperative that we understand the mechanisms that co-ordinate mitochondrial function with environmental signaling systems. The regulation of yeast mitochondrial function in response to nutritional change can be modulated by PKA activity. Unregulated PKA activity can lead to the production of mitochondria that are prone to the production of ROS, and an apoptotic form of cell death.

**Results:**

We present evidence that mitochondria are sensitive to the level of cAMP/PKA signaling and can respond by modulating levels of respiratory activity or committing to self execution. The inappropriate activation of one of the yeast PKA catalytic subunits, Tpk3p, is sufficient to commit cells to an apoptotic death through transcriptional changes that promote the production of dysfunctional, ROS producing mitochondria. Our data implies that cAMP/PKA regulation of mitochondrial function that promotes apoptosis engages the function of multiple transcription factors, including *HAP4*, *SOK2 *and *SCO1*.

**Conclusions:**

We propose that in yeast, as is the case in mammalian cells, mitochondrial function and biogenesis are controlled in response to environmental change by the concerted regulation of multiple transcription factors. The visualization of cAMP/*TPK3 *induced cell death within yeast colonies supports a model that PKA regulation plays a physiological role in coordinating respiratory function and cell death with nutritional status in budding yeast.

## Background

Mitochondria participate in a number of essential cellular functions, for example they are key players in ATP production, via the process of oxidative phosphorylation, which can produce up to 15 times more ATP from glucose than glycolysis alone. They are also central to metabolic regulation and facilitate diverse cell signaling events [1,2]. Mitochondria are therefore essential for the maintenance, adaptability and survival of eukaryotic cells. In addition, these remarkable organelles have been conclusively shown to play a role in the regulation of programmed cell death processes (reviewed in [3]), and act as an important determinants of cellular senescence and ageing [4,5]. The importance of understanding how mitochondria influence the metabolic status of cells becomes apparent when we consider that many muscular and neurodegenerative diseases have been linked with their dysfunction. The current list of disease pathologies in which mitochondrial function is thought to be a major contributing factor is extensive and includes diabetes [6], cancer [7], multiple sclerosis [8], Alzheimer's [9] and Parkinson's [10]. Good evidence also exists that indicates a loss of mitochondrial function occurs during the progression of normal ageing [11]. Mitochondrial dysfunction can lead to the production of reactive oxygen species (ROS), which are implicated in both ageing and apoptosis [12,13], presumably as a result of their ability to damage macromolecules.

The control of mitochondrial biogenesis is complex, requiring coordinated transcription of a large number of nuclear and mitochondria encoded genes. In addition, there must be concerted control of the synthesis, import, and incorporation of proteins and lipids to existing mitochondria alongside appropriate replication of the mitochondrial DNA (mtDNA). In mammalian cells a number of have been indentified that act to co-ordinate tissue specific mitochondrial biogenesis in the face of altered metabolic demand and environmental change, including; nuclear respiratory factor-1 (NRF-1) and GA-binding protein (GABP or NRF-2), peroxisome proliferator-activated receptors (PPARα, PPARδ, and PPARγ), Mitochondrial transcription factors TFAM, TFB1M and TFB2M, Estrogen-related receptors (ERRα, ERRβ, and ERRγ) (for a recent review see [14]). The mitochondria of yeast are also adapted to respond to rapidly changing metabolic demands. For example switching between fermentative and non-fermentative carbon sources requires adjustment of mitochondrial activity and density [15]. However despite the importance of the mitochondria to cellular function, the signaling mechanisms by which their activity is co-ordinated with environmental conditions in yeast are not well documented.

Previous studies in yeast have established links between Ras signaling and mitochondrial function, via cAMP/PKA dependent and independent routes [16,17]. Within yeast Ras/cAMP/PKA signaling also controls cellular processes that include cell growth and proliferation and the induction of stress responses [18], making this pathway a good candidate to integrate environmental signaling with mitochondrial regulation. In yeast, protein kinase A (PKA) consists of a single regulatory subunit encoded by the *BCY1 *gene [19] and three catalytic subunits Tpk1p, Tpk2p and Tpk3p [20]. PKA is activated by an increase in cAMP concentration, which is generated in the cell from adenylyl cyclase, Cyr1p [21]. The three catalytic subunits of yeast exhibit high similarity and have been shown to have both specific and overlapping functions. For instance Tpk2p has been shown to influence iron uptake, trehalase synthesis, water homeostasis [22] and pseudohyphal growth [23]. Tpk1p has been implicated in the branched chain amino acid biosynthesis pathway, mitochondrial iron homeostasis and mtDNA stability [22]. Excessive PKA activity can prove deleterious, for instance the overexpression of *TPK3 *has been shown to inhibit growth [24]. An important regulator of Cyr1p activation is the small regulatory GTPase Ras [21]. The regulation of cAMP production from Cyr1p in response to Ras activation also requires the protein Srv2p/CAP [25,26], which is able to bind both adenylyl cyclase and actin structures. We have shown previously that the accumulation of stable actin aggregates leads to the hyperactivation of the Ras/cAMP/PKA pathway [27]. The result of actin aggregation induced Ras/cAMP/PKA signaling is that ROS are produced from dysfunctional mitochondria, facilitating cell death that displays hallmarks of yeast apoptosis [27-29]. The deletion of *TPK3 *is sufficient to prevent the production of ROS in actin aggregating strains, implicating this PKA subunit as a regulator of mitochondrial function [27,30]. Further evidence for a role of Tpk3p in the regulation of mitochondrial function comes from a study in which cells lacking Tpk3p showed reduced respiratory activity [31].

Here we demonstrate that increased Tpk3p activity is sufficient to induce formation of dysfunctional mitochondria with striking morphological abnormalities that produce high levels of ROS. We provide evidence that the loss of mitochondria function and ROS production associated with elevated Tpk3p activity arises as a result of transcriptional changes that inhibit mitochondrial biogenesis, corrupt the electron transport chain and inhibit stress response mechanisms. We also show that the loss of mitochondrial function and production of ROS requires the activity of the transcriptional regulators *HAP4*, *SOK2 *and *SKO1*. This paper therefore establishes important links between cAMP/PKA signaling, nutritional sensing, mitochondrial biogenesis and ROS production.

## Methods

### Yeast strains, plasmids, Media and Growth Conditions

All yeast strains used in this work are derivatives of BY4741 and are listed in Table 1. Unless stated otherwise, cells were grown in a rotary shaker at 30°C in synthetic complete medium or synthetic medium lacking an appropriate nutrient for auxotrophic selection. *PDE2 *was disrupted using the loxP marker cassette system [32]. Oligonucleotides designed for the targeted disruption of genes are described in Additional File 1. Plasmids used to overexpress *TPK3 *[33] and *HAP4 *[34] have also been described previously.

**Table 1:** Yeast strains used in this study

Strain	Genotype	Origin
CGY424	mata *his3Δ1 leu2Δ met15Δ ura3Δ*	Research Genetics

CGY502	mata *his3Δ1 leu2Δ met15Δ ura3Δ Δpde2::KanMx*	Research Genetics

CGY217	mata *his3Δ1 leu2Δ met15Δ ura3Δ Δpde2::HIS3 Δtpk3::KanMx*	[27]

CGY215	mata *his3Δ1 leu2Δ met15Δ ura3Δ Δpde2::HIS3 Δtpk1::KanMx*	[27]

CGY216	mata *his3Δ1 leu2Δ met15Δ ura3Δ Δpde2::HIS3 Δtpk2::KanMx*	This study

CGY807	mata *his3Δ1 leu2Δ met15Δ ura3Δ Δpde2::HIS3 Δtpk1::KanMx Δtpk3::LEU2*	This study

CGY808	mata *his3Δ1 leu2Δ met15Δ ura3Δ Δpde2::HIS3 Δtpk2::KanMx Δtpk3::LEU2*	This study

CGY819	mata *his3Δ1 leu2Δ met15Δ ura3Δ Δpde2::HIS3 Δtpk2::KanMx Δtpk3::LEU2*	This study

CGY638	mata *his3Δ1 leu2Δ met15Δ ura3Δ Δcox4::KanMx*	This study

### RNA Isolation and Affymatrix Microarray Procedure

Strains CGY502 (*Δpde2*) and CGY217 (*Δpde2Δtpk3*) were grown for 24 hours in YPD (1% yeast extract, 2% Bacto-peptone, 2% glucose) supplemented with 4 mM cAMP at 30°C in a rotary air incubator. Cells from triplicate 20 ml cultures were harvested by centrifugation and resuspended in a small volume of media. The suspension was dispensed dropwise into liquid nitrogen. Triplicate cell pellets were maintained on dry ice and sent to the COGEME Transcriptome facility at the University of Manchester http://cogeme.ex.ac.uk/index.html for RNA isolation and microarray analysis. A yeast2 Affymetrix array was used in these experiments and statistical analysis on a gene by gene basis carried out using Limma software. Quality Control analysis was carried out using dchip software. Raw and normailsed data from the microarray has been deposited in the Gene Expression Omnibus (GEO) database, accession number GSE25541.

### High Resolution Respirometry

Intact cell respiration was determined at 30°C using an Oxygraph-2 k system (Oroboros, Innsbruck, Austria) equipped with two chambers. Data was analysed using DatLab software. Yeast cells (2 ml) at a concentration of 3.5 × 10^6^/ml, in minimal media without glucose, were added to each chamber. All assays were conducted in biological triplicate. The chambers were closed and Routine respiration was recorded. LEAK respiration was determined by the addition of 150 μM TET (Sigma), an ATP synthase inhibitor. Uncoupled respiration was then determined by the addition of the ionophore FCCP (12 μM) (Sigma). The addition of 2 μM Antimycin A (Sigma) accounted for non-mitochondrial oxygen consumption.

### Immunoblotting

Cell number was accurately determined using a haemocytometer and total protein extracted using as optimized protein extraction for method suitable for quantitative proteomics [35]. Samples were separated by SDS PAGE and transferred to PVDF membranes before probing with primary antibodies at the final concentrations, anti-Cox4 (mitoscience 1: 1000), anti-Cox2 (mitoscience 1:1000), anti-Por1 (Molecular Probes, Invitrogen 1:500) and anti-actin (1:2000).

### Fluorescence microscopy

Rhodamine-phalloidin and DAPI staining were performed as previously described for F-actin [36]. Cells were viewed with an Olympus IX-81 fluorescence microscope with a 150 W Xenon/mercury lamp and an Olympus 150× Plan NeoFluor oil-immersion objective. GFP/RFP Co-localisation studies were performed using an Optosplit II Image Splitter (Cairn Scientific). Images were captured using a Hammamatsu ORCA AG digital camera using Olympus Cell R software.

### Analysis of mitochondria

Assessment of reactive oxygen species content and mitochondrial membrane potential were carried out as previously described[27].

GFP labeled mitochondria were visualized using the plasmids pVTU100U-mtGFP and pYX122-mtGFP [37].

### Analysis of cell death in yeast colonies

Between 10 and 20 cells were plated onto 9 cm YPAD agar plates containing 10 μM Phloxine B (Sigma). Colonies were grown for five days before being visualized using a Leica M2FLIII microscope and documented with a Leica DC300F colour camera. To visualise a cross section through the colony, a glass coverslip was inserted directly, bisecting the colony, and removed from the agar. Dissected colonies were visualized and documented immediately. All images were taken using the same illumination conditions and exposure times.

### Glycogen staining

Iodine staining of yeast cells as an assay for glycogen content was carried out as previously described (Care et al., 2004).

## Results

### cAMP/PKA regulation of respiratory function

**Figure 1:**
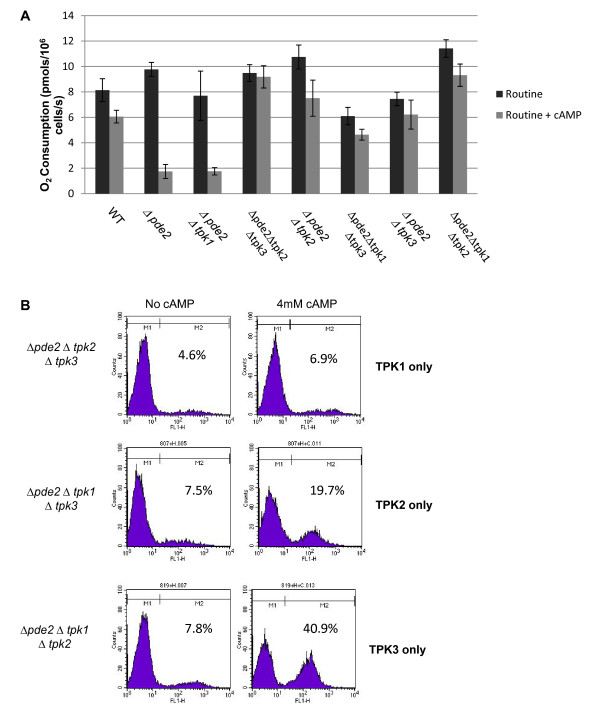
**Effects of PKA activity on respiratory function and ROS production**. We analysed the influence of individual subunits on respiratory function by measuring oxygen consumption in strains lacking combinations of *PKA *subunit gene deletions (A). The cAMP phosphodiesterase *PDE2 *was also deleted to allow elevation of cAMP levels by addition of exogenous cAMP (4 mM). ROS production in response to cAMP elevation was analysed in strains expressing only one of each of the three PKA subunits, Tpk1p, Tpk2p or Tpk3p (B). All strains were grown for 24 h to the diauxic phase of growth.

Our previous studies have shown that aggregation of the actin cytoskeleton results in hyperactivation of the Ras/cAMP/PKA signaling pathway, leading to the loss of respiratory function, ROS production and an apoptotic cell death [27]. The *S. cerevisiae *genome encodes for three PKA subunits, encoded by *TPK1*, *TPK2 *and *TPK3*. In order to establish which PKA subunits were required to induce the loss of respiration and ROS production, we generated a series of mutant strains expressing either a single PKA isoform, or combinations of multiple subunit encoding genes. Using this genetic approach we were able to assess the influence of individual PKA isoforms on respiratory function during normal growth, or under conditions of elevated cAMP. Elevation of cAMP levels was achieved by the deletion of the high affinity cAMP phosphodiesterase, *PDE2*, which catalyses the breakdown of cAMP. The loss of Pde2p function allows manipulation of levels of this secondary messenger by exogenous cAMP addition [38]. We performed high resolution respirometry on PKA mutant strains grown for 24 h to diauxic shift in the presence or absence of 4 mM cAMP, using an Oroboros Oxygraph, which accurately measures changes in oxygen concentration in a sealed system. By measuring the fall in oxygen concentration within the chamber the oxygen flux can be determined. Oxygen consumption, which occurs at the Cytochrome c Oxidase complex (complex IV), is a direct measure of electron transport system (ETS) activity. Using this sensitive technique it was possible to detect a significant reduction in respiratory activity in wild type cells grown in the presence of exogenous cAMP (Figure 1A). Interestingly, the loss of *PDE2 *led to a significant increase in respiration, while addition of further cAMP in this background led to a dramatic reduction in oxygen consumption. These results suggest that the level of respiratory activity is modulated in response to variation of intracellular cAMP levels.

The addition of cAMP to cells lacking both *PDE2 *and the *TPK1 *subunit genes led to a reduction in respiration similar to that observed in the *Δpde2 *single mutant. However while the level of routine respiration differed between strains (Figure 1A), triple mutant *Δpde2Δtpk2Δtpk3*, *Δpde2Δtpk1Δtpk3 *or *Δpde2Δtpk1Δtpk2 *strains did not exhibit the collapse of respiratory activity observed *Δpde2 or Δpde2Δ*tpk1 cells when cAMP levels were elevated (Figure 1A). Similarly *Δpde2Δ*tpk2 or *Δpde2Δtpk3 *double mutant cells were resistant to the effects of elevated cAMP. Collectively these data suggest that the collapse of respiration observed in *Δpde2 *cells when grown in the presence of exogenous cAMP requires the presence of both *TPK2 *and *TPK3*, but occurs independently of *TPK1*.

We then examined the accumulation of ROS in response to cAMP elevation within cell populations expressing Tpk1p, Tpk2p or Tpk3p as their sole source of PKA activity (Figure 1B). We found that the elevation of cAMP in strains expressing *TPK1 *only resulted in no difference in the number of cells producing high ROS levels within a population (Figure 1B). In contrast, cells expressing *TPK2 *or TPK3 only did show an increase in the number of ROS producing cells when cAMP levels were increased (Figure 1B). These data are in accordance with the findings in Figure 1A, that both *TPK2 *and *TPK3 *play a role in the regulation of respiratory activity in response to cAMP elevation. However it should be noted that cells expressing *TPK3 *only displayed the most significant increase in the number of ROS producing cells (Figure 1B), indicating that this PKA subunit may play a more fundamental role in the regulation of radical production.

### Tpk3p activity is sufficient to induce respiratory collapse and ROS production

**Figure 2:**
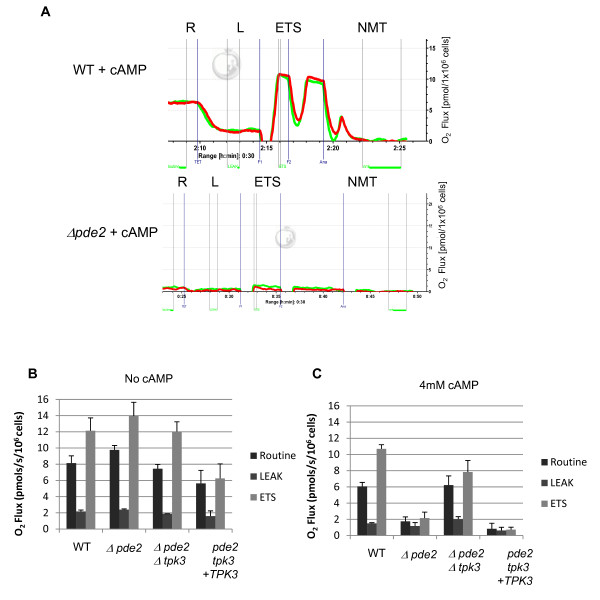
**Excessive Tpk3p activity leads to loss of mitochondrial respiration**. Cells were grown for 24 hours to diauxic shift in minimal medium in the presence or absence of 4 mM cAMP. Mitochondrial respiration was measured using an Oroboros high resolution respirometer. Typical traces from two biological replicates (red and green lines) wild type and *Δpde2 *cells grown in the presence of 4 mM cAMP are shown (A). Routine respiration (designated by R) represents oxygen consumption from cells placed in the respirometer immediately from culture and without drugs. The addition of TET, an ATPsynthase inhibitor, causes proton build up on the outside of the inner mitochondrial membrane, inhibiting respiration. Respiration that does occur happens as a result of the small LEAK of protons across the inner membrane (designated by L). Dissipation of this proton gradient by the proton ionophore FCCP restores mitochondrial respiration (designated by ETS) but removes the major control point, being the proton gradient which is controlled by the rate of oxidative phosphorylation. The final addition of Antimycin A blocks activity of complex III, preventing electrons moving to complex IV and therefore oxygen consumption is halted and non-mitochondrial oxygen consumption (designated by NMT) is accounted for. The oxygen consumption under these conditions was determined for strains wild type, Δpde2, Δpde2 Δtpk3 and Δpde2Δtpk3 + *TPK3 *grown in the absence (B) and presence (C) of 4 mM cAMP. Error bars are the standard error of 3 independent experiments.

Although *TPK2 *and *TPK3 *would appear to be essential to induce the collapse of respiration under conditions of elevated cAMP (Figure 1A), our previous data points to the loss of Tpk3p as sufficient to prevent ROS production from the mitochondria when cAMP levels are raised [27]. We therefore sought to characterise in detail the mitochondrial defects associated with elevated Tpk3p activity. Wild type, *Δpde2*, *Δpde2 Δtpk3 *and *Δpde2 Δtpk3 *+ *TPK3 *strains were cultured for 24 hours in media supplemented with 2% glucose to the diauxic shift phase of growth. During the diauxic shift cells sense a reduction in glucose and prepare to shift to a non-fermentative metabolism, which requires functional mitochondria. At this stage the basal, or routine, level of respiration was assessed (Figure 2A, marked R). Following stabilisation of routine respiration, the ATPsynthase/Complex V inhibitor, triethyltin bromide (TET) was used to determine LEAK flux (Figure 2A. marked L) which represents non-phosphorylating respiration due to proton leakage back across the Inner Mitochondrial Membrane. Oligomycin is commonly used for this purpose [39] but we found that the BY4741 background was resistant to its effects. TET can also be utilised to inhibit complex V [40] and following titration studies a concentration of 150 μM was found to be effective for this purpose in our strain background. Following treatment with TET, cells were exposed to 12 μM FCCP, a protonophore, which is able to dissipate the mitochondrial membrane potential. The proton gradient is a major control point of the ETS and its removal allows quantification of the maximum capacity of the ETS (Figure 2A. marked ETS). A second dose of 3 μM FCCP (seen as a second peak Figure 2A.) was administered to demonstrate that 12 μM FCCP was sufficient for complete uncoupling and the induction of maximal respiration. Finally, cells were exposed to the complex III inhibitor Antimycin A which prevents all mitochondrial related oxygen consumption (Figure 2A marked NMT). This allows quantification of NMT mediated respiration for subtraction from all data. However in our experiments no oxygen consumption was recorded in this respiratory state, indicating that no non-mitochondrial oxygen consumption was detectable in our system using this strain background.

Typical respirometry profiles for wild type and *Δpde2 *cells cultured in the presence of 4 mM cAMP are presented (Figure 2A). Significant differences were apparent between the respirometry profiles obtained for wild type and *Δpde2 *strains when grown in the presence of exogenous cAMP. While wild type cells typically demonstrated a routine respiration of around 6 pmols/s/10^6 ^cells under these conditions, cells lacking *PDE2 *showed a significant reduction, typically less than 2 pmols/s/10^6 ^cells (Figure 2A). This suggests that high levels of cAMP signaling suppress the respiratory activity of yeast mitochondria. To investigate this further we examined the respirometry profiles of wild type, *Δpde2, Δpde2Δtpk3 *and *Δpde2Δtpk3 *+ *TPK3 *grown in the presence or absence of cAMP (Figure 2B and summarised in Additional File 2). When cultured in the absence of cAMP both wild type and the double mutant *(Δpde2Δtpk3) *displayed similar routine respiration rates and maximal ETS levels upon FCCP addition. Interestingly the single mutant, *Δpde2*, showed a 20% increase in routine respiration and a maximal ETS that appeared higher than wild type when grown in the absence of additional cAMP (Figure 2B). Overexpression of *TPK3 *from a plasmid *in Δpde2Δtpk3 *resulted in a significant reduction in routine respiration, and notably cells failed to significantly increase respiration rate upon FCCP addition. In all strains the LEAK level of respiration appeared to be constant (Figure 2B).

The addition of 4 mM cAMP did appear to have a small effect on respiratory activity in wild type cells using this highly sensitive assay. We observed a 17% reduction in routine respiration when compared to wild type cells cultured without cAMP. A similar reduction was observed in the LEAK level of respiration, but the maximal ETS level remained unchanged. When cells lacking *PDE2 *were grown in the presence of exogenous cAMP we consistently observed a dramatic fall in routine respiration and maximal ETS rate (Figure 2C). Confirmation that Tpkp3 was responsible was obtained as respiratory activity was largely restored in *Δpde2Δtpk3 *cells grown under the same conditions (Figure 2C). Further evidence was obtained by the re-expression of *TPK3 *in *Δpde2Δtpk3 *cells which resulted in the loss of respiration in the presence of exogenous cAMP. In addition, the addition of FCCP did not elevate respiration in either Δ*pde2*Δ*tpk3 *or Δ*pde2*Δ*tpk3*+*TPK3 *cells, pointing to a loss of proton gradient across the inner mitochondrial membrane, further supporting the notion that elevated Tpkp3 activity leads to a loss of mitochondrial membrane potential. The data provided may also suggest that elevated levels of Tpk3p activity leads to reduction in the levels of complete and functional electron transport complexes.

### Effects of *TPK3 *on ROS production and mitochondrial morphology

**Figure 3:**
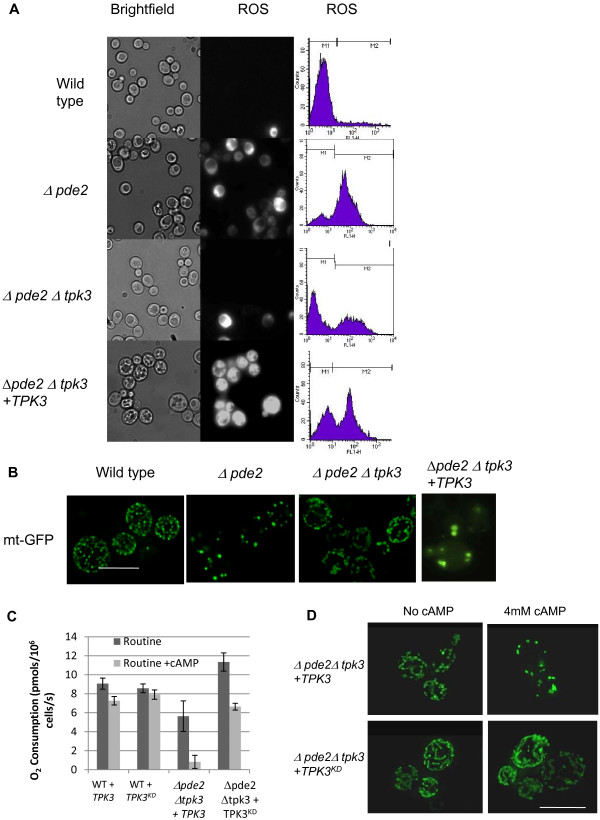
**Tpk3p dependent ROS production and mitochondrial dysfunction**. ROS production and aberrant mitochondrial morphology are a result of Tpk3p activity. We examined the effect on ROS production on diauxic shift cultures with and without high Tpk3p activity. Strains were grown in the presence of 4 mM cAMP for 24 hours in MM supplemented with H_2_DCF-DA. Cells were then either examined by fluorescence microscopy or flow cytometry for the presence of ROS, fluorescing cells that indicate the presence of ROS are found in the M2 region of the histograms presented (A). Mitochondrial morphology was investigated by expression of a targeted GFP molecule (B). Routine respiration was assessed in wild type and *Δpde2Δtpk3 *cells overexpressing an active *TPK3 *or a kinase dead version (*TPK3^KD^*) of the enzyme in the presence and absence of exogenous 4 mM cAMP (C). Mitochondrial morphology was also assessed in *Δpde2Δtpk3 *cells overexpressing *TPK3 *or *TPK3^KD ^*in the presence and absence of exogenous 4 mM cAMP (D). Scale Bar = 10 μM.

To establish whether elevated Tpk3p activity was sufficient to induce ROS production, wild type, *Δpde2*, *Δpde2 Δtpk3 *and *Δpde2 Δtpk3 *+ *TPK3 *cultures were grown in the presence of the fluorescent indicator dye H_2_DCF-DA. Samples were anlaysed by flow cytometry as well as visually under a fluorescent microscope (Figure 3A). In the presence of cAMP wild type cells were found to be generally unaffected, presumably as they were able to breakdown additional cAMP (data not shown). However the loss of Pde2p, and hence the ability to efficiently break down cAMP, resulted in a significant increase in ROS accumulating cells (Figure 3A). The additional loss of *TPK3 *in cells lacking *PDE2 *resulted in a significant reduction in the number of ROS positive population (Figure 3A). Importantly the re-expression of *TPK3 *on a plasmid in *Δpde2 Δtpk3 *cells restored production of ROS (Figure 3A).

In order to investigate the effects of Tpk3p on mitochondrial morphology we made use of a plasmid that allows the expression of a mitochondrial targeted RFP protein (a kind gift from J. Shaw University of Utah) (Figure 3B). While a reticular network was seen in wild-type cells, mitochondria in *Δpde2 *mutant cells appeared reduced in number, were fragmented and visibly enlarged when grown in the presence of exogenous cAMP (Figure 3B). The abnormal mitochondria observed could be restored to a wild type appearance in cells lacking both *PDE2 *and *TPK3 *(Figure 3B). We also noted that in the *Δpde2 *mutant only a few enlarged structures of mtDNA could be seen when compared to the numerous structures visible in wild type (data not shown). In line with the improvement in mitochondrial morphology, the number of mt nucleoids was increased in *Δpde2Δtpk3 *cells (data not shown). In addition fragmentation of genomic DNA (gDNA) was apparent in *Δpde2 *cells, a phenotype indicative of apoptosis. In contrast nuclear DNA fragmentation did not appear in *Δpde2Δtpk3 *cells (data not shown), consistent with our previous reports that Tpk3p induced mitochondrial dysfunction can trigger an apoptotic form of cell death [27,30]. In line with the ROS accumulation result (Figure 3A), the re-expression of *TPK3 *in *Δpde2Δtpk3 *was sufficient to restore the highly fragmented mitochondrial appearance observed in *Δpde2 *cells.

To confirm that the kinase activity of Tpk3p is responsible for the respiratory and morphological defects observed in *Dpde2 *cells when cAMP levels are elevated we generated a kinase dead version of the enzyme. Deminoff *et al *(2006) demonstrated that the mutagenesis of residues K336 and H338 to alanine in the yeast PKA, Tpk1p produced a protein lacking kinase activity. As the catalytic domain of *TPK1 *and *TPK3 *are highly conserved, we mutagenised the equivalent K337 and H339 residues of Tpk3p to alanine. In line with these mutations rendering Tpk3p inactive, the expression of *TPK3^KD ^*in *Dpde2Dtpk3 *cells did not lead to the collapse of respiration (Figure 3C) or to the manifestation of a highly fragmented mitochondrial network (Figure 3D). These data further suggest that elevated kinase activity of Tpk3p is sufficient to induce respiratory collapse and ROS production in yeast mitochondria.

### cAMP/Tpk3p Signalling regulates cell death in yeast colonies

**Figure 4:**
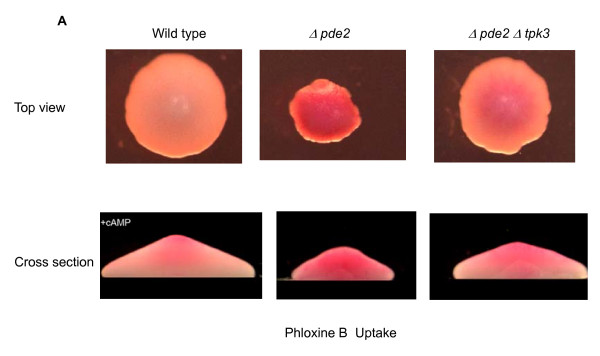
**Elevated Tpk3p activity affects colony morphology**. Cell death was visualized within growing yeast colonies grown for 5 days on rich media containing 10 μM Phloxine B containing 4 mM cAMP. Cells that are dead or dying accumulate the dye which stains them pink/red. Colonies were visualised from above before dissection and images captured incross section.

It has recently emerged that regulated cell death is employed within yeast colonies to facilitate multi-cellular communication and simple differentiation [41,42]. We therefore assessed the effects of Tpk3p activity on the patterning of cell death within colonies. We grew colonies for five days on YPD medium supplemented with 4 mM cAMP and the cell death indicator Phloxine B, which accumulates within dead or dying cells [27]. Wild type cells of this background display an accumulation of Phloxine B within the centre of the colony, which is most visible in the dissected cross section view presented in Figure 4. Colonies grown from cells lacking *PDE2 *were smaller, disordered and accumulated phloxine B throughout the colony, indicating widespread death. Small regions of phloxine B exclusion were apparent at the very growing edge of the colony. The deletion of *TPK3 *in *Δpde2 *cells resulted in a restoration of colony size and a central staining pattern that was slightly enlarged when compared to wild type but which represented a significant rescue of the cAMP induced colony death phenotype observed in single mutant *Δpde2 *colonies. These data suggest that cAMP/PKA signaling regulation of cell death plays a significant role in yeast colony patterning.

### Microarray analysis of changes in gene expression mediated by Tpk3p activity

**Figure 5:**
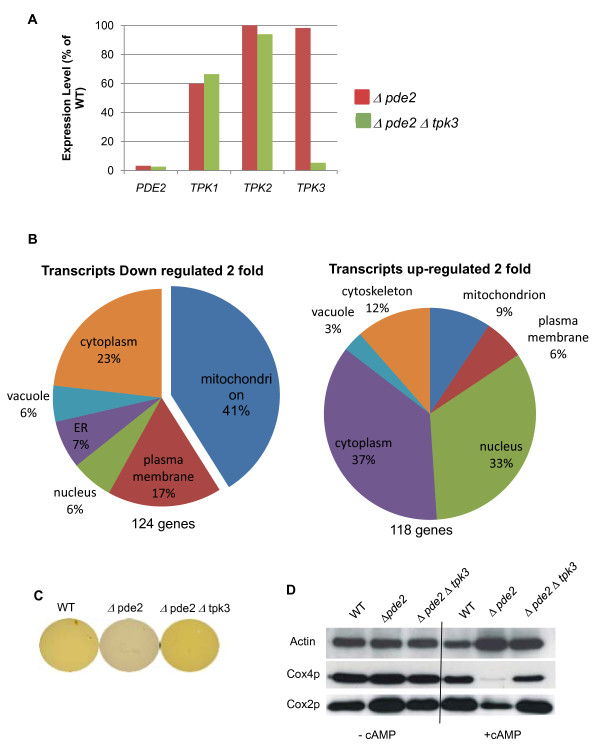
**Microarray analysis of the effects of elevated Tpk3p activity**. The effect of high Tpk3p activity on gene transcription was assessed. An affymatrix microarray was performed on RNA isolated from *Δpde2 *and *Δpde2 Δtpk3 *strains grown to diaxuic shift in YPD media supplemented with 4 mM cAMP. Confirmation of both the strains genotype and reliability of the micro array data can be seen by comparing expression levels of the effected genes *PDE2 *and *TPK3: TPK1 *and *TPK2 *expression levels are included for information (A). GO-Slim Mapper software was used to identify likely intracellular compartments of genes either up or down regulated by at least 2 fold (B). Genes involved in glycogen biosynthesis were significantly down regulated in *Δpde2 *cells under these conditions but not in *Δpde2 Δtpk3*. As further verification of the array data we assayed glycogen levels in wild type, *Δpde2 *and *Δpde2 Δtpk3 *cells. In this assay a yellow colour is indicative of glycogen presence (C). Western blotting was used to assess the levels of the mitochondrial Cytochrome c Oxidase protein Cox4p, Cox2p and actin (Act1p) in wild type, *Δpde2 *and *Δpde2 Δtpk3 *cells grown in the presence or absence of 4 mM cAMP. Protein equivalent to 2 × 10^6 ^cells was run on an SDS-PAGE gel and transferred to PVDF for immunodetection (D).

Our data indicate that elevated levels of Tpk3p activity have a profound effect on the respiratory capacity and function of yeast mitochondria. In an attempt to identify the regulatory mechanisms involved we conducted a microarray experiment. Single mutant *Δpde2 *and double mutant *Δpde2 Δtpk3 *strains were grown to diauxic shift in the presence of 4 mM cAMP, and mRNA levels analysed as described in materials and methods. Comparison of the data sets obtained allowed us to assess the impact of Tpk3p activity on transcriptional regulation. Two natural internal controls within the microarray data, namely the deletion of *PDE2 *and *TPK3 *in the single and double mutants compared to the wild type, allowed us to confirm the reliability of the data obtained. Expression of *PDE2 *in both the single and double mutant was 3.3% and 2.7% respectively compared to the wild type; *TPK3 *expression levels were 98% in the single *Δpde2 *mutant and 5.3% in the *Δpde2Δtpk3 *double mutant, confirming the genotype of the strains used in this study (Figure 5A). Interestingly expression levels of *TPK2 *were virtually unchanged in either mutant (105% - Δ*pde2 *and 94% - Δ*pde2 *Δ*tpk3*) whereas *TPK1 *levels were reduced to 60% of wild type levels in Δ*pde2 *and 66% in the double mutant. In line with this Tpk1p protein levels were also markedly reduced in *Δpde2 *cells when grown in the presence of exogenous cAMP (data not shown). Of the 5800 transcripts screened, 118 were up-regulated by at least 2 fold and 124 down-regulated at least 2 fold when comparing *Δpde2 *and *Δpde2Δtpk3 *strains (listed in Additional files 3 and 4). Gene ontology anaylsis, using the GO-Slim Mapper software http://www.yeastgenome.org/cgi-bin/GO/goSlimMapper.pl, allowed allocation of these genes to intracellular compartments (Figure 5B). We found that 41% of the down-regulated transcripts encoded mitochondrial genes. Closer inspection of the down-regulated mitochondrial genes revealed a number of interesting targets that are involved directly in the mitochondrial ETS (Table 2). Both external mitochondrial NADH dehydrogenases, *NDE1 *and *NDE2*, which represent Complex I in yeast, were down regulated by 2 and 5 fold respectively. Similarly *SDH2*, a component of complex II - succinate dehydrogenase; *CYT1 *and *RIP1 *complex III subunits; *CYC1-*isoform-1 cytochrome c; *COX4 *a subunit of complex IV - Cytochrome c Oxidase and *ATP19 *a component of ATPsynthase were all down regulated. In addition, the expression of a number of regulatory components of the ETS was also reduced, including *COQ2*, which is required for ubiquinone biosynthesis; *CYT2*, an enzyme required for cytochrome c maturation; *COX10 *and *SCO1*, both required for Cytochrome c Oxidase formation and *NCA3 *which regulates expression of the mitochondrially encoded ATP synthase subunits 6 and 8. In addition to the loss of crucial elements of the ETS the microarray revealed ROS detoxification enzymes (*SOD1, SOD2 *and *CTT1*) were not transcriptionally up-regulated despite the increase in ROS (Table 2).

**Table 2:** Selected genes downregulated by elevated cAMP/*TPK3 *signalling

Function	Gene	Fold Change	Gene Function
PKA	*TPK1*	-1.1	PKA catalytic subunit
	*TPK2*	1.1	PKA catalytic subunit

ETS	*NDE1*	-2.0	Mitochondrial external NADH dehydrogenase, catalyzes the oxidation of cytosolic NADH;
	*NDE2*	-5.5	Mitochondrial external NADH dehydrogenase, catalyzes the oxidation of cytosolic NADH;
	*SDH2*	-2.3	Iron-sulfur protein subunit of succinate dehydrogenase Complex II
	*COQ2*	-2.0	Catalyzes the second step in ubiquinone (coenzyme Q) biosynthesis
	*RIP1*	-1.7	Rieske Iron Sulphur Protein of Complex III
	*CYT1*	-2.0	Cytochrome c1, catalytic subunit of complex III
	*CYT2*	-2.3	Cytochrome c1 heme lyase, involved in maturation of CYT1
	*CYC1*	-1.9	Cytochrome c, isoform 1;transfers electrons from Complex III to Complex IV
	*COX4*	-2.2	Subunit IV of cytochrome c oxidase (Complex IV)
	*COX10*	-2.3	Heme A:farnesyltransferase, required for cytochrome c oxidase activity;
	*SCO1*	-2.0	Copper-binding protein of the mitochondrial inner membrane, required for cytochrome c oxidase
	*NCA3*	-6.2	Regulates mitochondrial expression of subunits 6 (Atp6p) and 8 (Atp8p) of Complex V
	*ATP19*	-2.2	Subunit k of the mitochondrial F1F0 ATP synthase

Detox	*SOD1*	-1.4	Cytosolic superoxide dismutase
	*SOD2*	-1.1	Mitochondrial superoxide dismutase; protects cells against oxygen toxicity
	*CTT1*	-1.6	Cytosolic catalase T, has a role in protection from oxidative damage by hydrogen peroxide

Selected genes involved in ETS and ROS detoxification that were downregulated in *Δpde2 *cells but not in *Δpde2 Δtpk3 *when grown in the presence of exogenous 4 mM cAMP. Gene lists were obtained as a ratio of statistically significant expression levels in *Δpde2*/*Δpde2 Δtpk3 *cells when grown in the presence of 4 mM cAMP.

Examination of up-regulated genes did not reveal any components of the ETS However genes involved in cellular respiration and mitochondrial regulation were identified (Table 3). These include *PUF3*, which links mitochondria to actin cables and is important for inheritance and morphological regulation [43]. Certain genes associated with cellular stress response were also up-regulated in a Tpk3p dependent manner. For example an 8 fold increase in transcripts encoding the DNA damage response gene *HUG1 *was found. Interestingly, several transporters involved in glucose uptake, *HXT1*, *HXT2 *and *HXT11 *were also upregulated (Table 3 and Additional File 3). These transporters are induced when the cell senses high glucose levels. Another gene that supported the idea that nutrient sensing in terms of glucose was compromised included *MIG2 *which has been shown to inhibit *SUC2 *expression in the presence of high glucose levels (Table 3).

**Table 3:** Selected genes uppregulated by elevated cAMP/*TPK3 *signalling

Function	Gene	Fold Change	Gene Function
DNA damage	*HUG1*	8.0	Responds to DNA damage or replication arrest, transcription is induced by DNA damage
	*THI4*	3.4	Thiazole synthase, required for thiamine biosynthesis and for mitochondrial genome stability

Nutrient sensing	*HXT1*	8.0	Glucose transporter, expressed in the presence of glucose, repressed when glucose is limited
	*MIG2*	5.0	Supressor of SUC2 expressed under high glucose levels

Mitochondrial Function	*PUF3*	2.4	OMM protein, linksArp2/3 complex with the mitochore promotes degradation of mRNAs for select nuclear-encoded mitochondrial proteins
	*AAC3*	3.4	IMM ADP/ATP translocator, exchanges cytosolic ADP for mitochondrially synthesized ATP; roles in maintenance of viability and in respiration

Genes involved in mitochondrial function, DNA damage response and nutrient sensing that were upregulated in *Δpde2 *cells but not in *Δpde2 Δtpk3 *when grown in the presence of exogenous cAMP. Gene lists were obtained as a ratio of statistically significant expression levels in *Δpde2*/*Δpde2 Δtpk3 *cells when grown in the presence of 4 mM cAMP.

A known feature of PKA activity is the ability to suppress the activation of biosynthesis of the storage carbohydrates glycogen and trehalose. This effect was noted in our array data. Several genes whose products are involved in glycogen and trehalose biosynthesis were downregulated in a Tpk3p dependent manner (Additional File 5). As a further control to verify the validity of our microarray data we investigated whether elevated Tpkp3 activity resulted in the suppression of glycogen accumulation as cells enter the stationary phase of growth. Wild type, *Δpde2 *and *Δpde2 Δtpk3 *cells were grown for 48 h and stained for glycogen accumulation as described in materials and methods. Wild type cells start to accumulate glycogen at this point of growth (Figure 5C), however we found that cells lacking *PDE2 *fail to accumulate glycogen. This failure to accumulate glycogen could be reversed by the additional deletion of *TPK3 *(Figure 5C).

The microarray data also suggested that the elevation of cAMP levels leds to a Tpk3p dependent reduction in *COX4 *transcript levels, a core component of Cytochrome c Oxidase. We tested whether this observation could be re-capitulated at the protein level by conducting a western blot using wild type, *Δpde2 *and *Δpde2Δtpk3 *strains were grown to diauxic shift in the absence or presence of 4 mM cAMP. In all strains grown without the addition of exogenous cAMP there appeared to be similar levels of Cox4p (Figure 5D). However upon addition of cAMP Cox4p was barely detectable in *Δpde2 *cells and a partial restoration was observed in *Δpde2Δtpk3 *cells. As Cox4p is essential for complex IV stability, these data suggested that high levels of cAMP and Tpk3p activity may lead to a loss or respiration as a result of the loss of Cytochrome c oxidase from the mitochondria. In line with this the level of Cox2p, which is encoded by the mitochondrial genome, was also greatly reduced upon cAMP elevation in *Δpde2 *cells (Figure 5D).

### cAMP/PKA signaling regulates mitochondrial function and cell death via transcription factor activity

**Figure 6:**
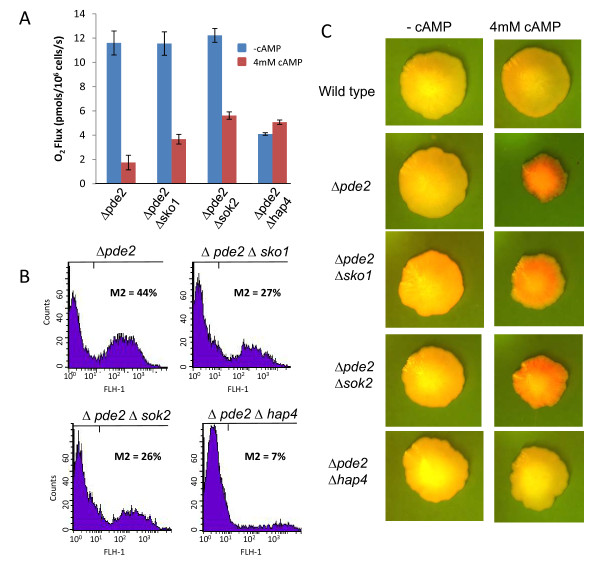
**Influence of transcription factors predicted to play a role in *TPK3 *based transcriptional remodeling under conditions of elevated cAMP**. Cells lacking *PDE2 *and either of the transcription factors *SKO1*, *SOK2 *or *HAP4 *were examined for their capacity to regulate routine respiration, ROS production and cell death in response to cAMP addition. Routine respiration was measured in *Δpde2*, *Δpde2Δsko1, Δpde2Δsok2, Δpde2Δhap4 *cells using a high resolution respirometer after growth for 24 h in YPD + 4 mM cAMP (A). The same strains and growth conditions were used to assess the production of ROS using H_2_DCF-DA (B) and the extent of cell death within colonies using phloxine B uptake as described in materials and methods (C).

Our microarray data suggest that elevated *TPK3 *activity leads to considerable remodeling at the level of transcription, with particular emphasis on genes involved in mitochondrial function. In an attempt to identify transcription factors involved we utilised the bioinformatics analysis package Yeastract http://www.yeastract.com/ to identify potential transcription factor involvement. Using this analysis software, which utilizes 48,333 characterised associations between known transcription factors and target genes, several candidate transcription factors were selected for further analysis, these were *SOK2*, *SKO1 *and *HAP4 *(Figure 6). The collapse of respiration observed in cells lacking *PDE2*, when grown in the presence of 4 mM cAMP, was partially rescued by the deletion of either *SKO1 *or *SOK2 *(Figure 6A). However in both cases a significant reduction in routine respiration rate was observed in response to cAMP addition. In contrast, although exhibiting a low starting routine rate of respiration, *Δpde2Δhap4 *cells did not respond to the addition of cAMP (Figure 6A). In line with a partial restoration of mitochondrial function, both *Δpde2Δsko1 and Δpde2Δsok2 *showed a significant reduction in the number of cells accumulating high levels of ROS when compared to *Δpde2 *single mutant cells (Figure 6B). Strikingly the addition of cAMP to *Δpde2Δhap4 *did not induce significant ROS production (Figure 6B). We also examined the effects of cAMP elevation on cell death in colonies in these strains (Figure 6C). We found that the loss of *SKO1 *or *SOK2 *in a *Δpde2 *background renders cells partially unresponsive to cAMP addition, resulting in increased colony size and reduced cell death as assessed by Phloxine B uptake (Figure 6C). Cells lacking both *PDE2 *and *HAP4 *appeared to be resistant to cAMP exposure and did not exhibit signs of widespread cell death (Figure 6C).

### *HAP4 *activity leads to the cAMP/PKA dependent biogenesis of dysfunctional mitochondria prone to ROS production

**Figure 7:**
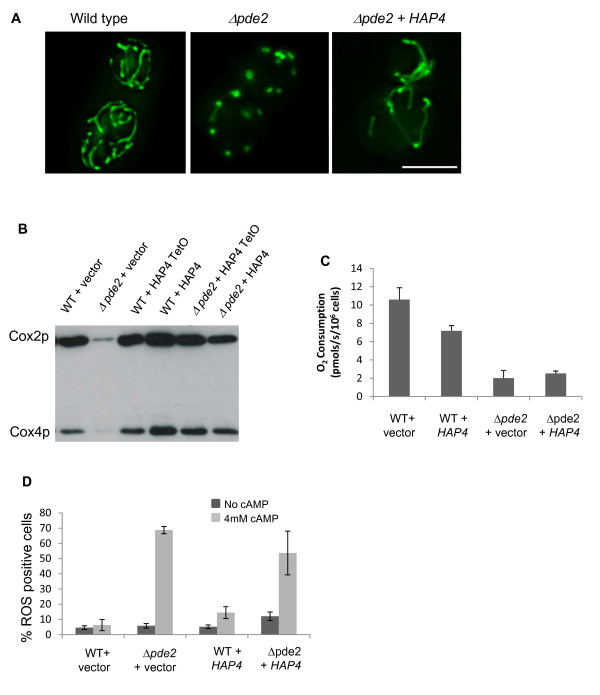
***HAP4 *overexpression leads to the production of dysfunctional mitochondria prone to ROS production**. Mitochondrial morphology was examined in wild type + empty plasmid, *Δpde2 *+ empty plasmid and *Δpde2 + HAP4 *cells grown to diauxic shift in minimal media supplemented with 4 mM cAMP using a mitochondria targeted GFP probe (A). The expression of nuclear and mitochondria encoded components of the Cytochrome c Oxidase complex (complex IV) was examined in wild type and *Δpde2 *cells grown in the presence of 4 mM cAMP (B). The influence of *HAP4 *overexpression on the ability of wild type and *Δpde2 *cells to respire (C) and to produce ROS (D) was examined under the same growth conditions. Scale Bar = 10 μM.

Our data points to a mechanism whereby cAMP induced ROS production and cell death occurs as a result of the modulation of multiple transcription factor activity. However the activity of *HAP4 *would appear to be of particular importance. It has been demonstrated that the *HAP4 *gene product is involved in stimulating mitochondrial biogenesis and elevating respiration during glucose de-repression, by enhancing the transcriptional activity of the HAP2/3/4/5 complex [44]. We therefore investigated the effects of *HAP4 *overexpression on mitochondrial function in wild type and *Δpde2 *cells grown in the presence of cAMP. To do this we overexpressed *HAP4 *from plasmids that utilized either an endogenous promoter or repressible Tetracycline, Tet O, promoter. Both of these plasmids were able to fully restore the inability of a *HAP4 *deletion strain to grow on the non-fermentable carbon source glycerol, indicating that both could restore mitochondrial function (data not shown). Initially we examined the ability of *HAP4 *overexpression to suppress the mitochondrial morphology defects associated with high PKA activity. As both plasmids yielded exactly the same results we present data for a single HAP4 expressing plasmid, that utilizing the Tet O promoter (Figure 7A). Mitochondria in cells lacking Pde2p develop fragmented and swollen mitochondria that fail to respire when grown in the presence of cAMP (Figure 7A and 3B). The overexpression of *HAP4 *was found to partially restore mitochondrial morphology to *Δpde2 *cells, in that a tubular array could be observed. However this network was never observed to be as elaborate as that seen in wild type cells (Figure 7A). The overexpression of *HAP4 *in *Δpde2 *cells was also able to increase the expression of nuclear (Cox4p) and mitochondrial encoded (Cox2p) components of the Cytochrome c Oxidase complex (complex IV) of the electron transport chain (Figure 7B). This indicates that expression of *HAP4 *is able to promote mitochondrial biogenesis in *Δpde2 *cells grown in the presence of cAMP. However the overexpression of *HAP4 *was insufficient to improve the poor oxygen consumption level observed in *Δpde2 *cells when treated with cAMP (Figure 7B). In addition, only a small reduction in the number of cells accumulating ROS could be observed when comparing *Δpde2 *cell grown in the presence of cAMP in the presence or absence of the *HAP4 *overexpression construct (Figure 7C). It should be noted that the overexpression of *HAP4 *in wild type cells also resulted in a significant reduction in oxygen consumption (Figure 7B) and an increase in the number or ROS positive cells (Figure 7C). These results suggest that although increased expression of *HAP4 *can lead to an increase in mitochondrial biogenesis, it does not by itself lead to the production of healthy and fully functional organelles.

## Discussion

The activity of cAMP/PKA signalling pathways appears to play an important role in the regulation of cell death in eukaryotes. For example it has been shown in lymphoid cells that elevated cAMP/PKA levels cause transcriptional changes that promote mitochondrial dependent apoptosis [45] andcan induce apoptosis via the up-regulation of Smac/DIABLO in cultured HeLa cells [46]. Our previous studies suggest that in yeast excessive PKA activity can also trigger, albeit via distinct signalling mechanisms to those observed to date in mammalian systems, mitochondria dependent apoptosis [27,28,30]. In yeast cAMP/PKA stimulated apoptosis involves the production of ROS from mitochondria. The mitochondrial state that leads to ROS production in actin aggregating cells requires PKA activity, and in particular the function of one of the three PKA subunits found in *S. cerevisiae*, Tpk3p [27,30]. Our data presented here supports a role for PKA signalling in regulating the activity of mitochondria via transcriptional control. In support of this our microarray analysis of the effects of cAMP elevation revealed a Tpk3p dependent suppression of a significant number of genes involved in mitochondrial function. Of particular note were genes whose products contribute to the electron transport system. The Tpk3p dependent downregulation of genes required for respiration also resulted in a significant reduction in respiratory capacity. Both the regulation of transcription and respiration could be restored by deletion of *TPK3*, implicating this single enzyme as an important regulator of mitochondrial biogenesis. Another possibility that must be considered is that Tpk3p localises to the mitochondria and regulates function directly via phosphorylation of protein targets. PKA activity within the mitochondria has been widely reported in mammalian systems and is known to modulate the activity of the respiratory chain [47]. Interestingly a fully functional carbon dioxide sensing cAMP/PKA signalling system has recently been identified within human HeLa and COS-1 cell lines [48]. In yeast, the mitochondrial histone-like protein Abf2 has been shown to be phosphorylated by PKA, suggesting that activity of cAMP dependent phosphorylation also occurs within mitochondria of lower eukaryotes [49]. Phosphorylation of Abf2p by PKA was shown to inhibit its ability to interact with DNA, and the loss of *ABF2 *resulted in mtDNA instability [49]. However fluorescence microscopy studies suggest that Tpk1p, Tpk2p and Tpk3p do not co-localise with the mitochondria (our unpublished results). In addition to this, the published mitochondrial proteome from *S. cerevisae*, despite identifying a number of protein kinases within the organelle, did not reveal the presence of PKA enzymes [50]. Further studies are required to fully address whether, as is the case in mammalian cells, cAMP/PKA signalling occurs within yeast mitochondria.

Further evidence that cAMP/Tpk3 signalling regulates mitochondrial biogenesis at the level of transcription comes from our finding that respiratory function, ROS production and cell death can be manipulated by rescued to varying degrees by the transcription factors *SOK2*, *SKO1 *and *HAP4*, which were predicted to play a role via bioinformatic analysis of our microarray data. Both *SOK2 *and *SKO1 *are transcription factors that have been previously implicated in cAMP signalling systems, and act as suppressor elements within stress signalling pathways. *SOK2 *is known to be regulated by cAMP/PKA activity [51] and to play an important role in the regulation of cell death in colonies in response to ammonia signalling [42]. *SKO1 *is a repressor that mediates HOG pathway-dependent regulation by binding to cAMP response elements (CRE) sites in target promoters [52]. It is also known to play a regulatory role within cAMP/PKA stress signalling and can be phosphorylated by both *HOG1 *and PKA at independent sites [53]. It is likely that some of the toxicity associated with elevated cAMP/PKA activity occurs as a result of increased stress response suppression via the activity of *SOK2 *and *SKO1*. In line with this, the deletion of either *SOK2 *or *SKO1 *led to a partial restoration of respiratory activity and reduction in ROS production in *Δpde2 *cells grown in the presence of cAMP.

More significantly, the deletion of *HAP4*, a known mitochondrial biogenesis factor [54], prevented ROS production and cell death in *Δpde2 *cells grown in the presence of cAMP. This suggests that the activity of *HAP4 *present within cells experiencing high cAMP/PKA signalling is responsible for the production of ROS. The overexpression of *HAP4 *led to the partial restoration of mitochondrial biogenesis in *Δpde2 *cells grown under conditions of cAMP elevation. However the additional mitochondria produced were not competent to respire and continued to produce high levels of ROS. We found that the overexpression of *HAP4 *in wild type cells also gave rise to mitochondria with reduced respiratory capacity, which were prone to ROS production. These data suggest that as yet unidentified factors aside from *HAP4 *are required to ensure the biogenesis of fully functional mitochondria. As the loss of *TPK3 *led to the restoration of fully functional mitochondria in *Δpde2 *cells grown under conditions of cAMP elevation it is likely that PKA function regulates mitochondrial activity via more than one effector to co-ordinate biogenesis in response to environmental change and metabolic demand. The search for further downstream targets of Tpk3p that are involved in mitochondrial regulation and biogenesis is currently underway. Elevation of Tpk3p activity also led to striking morphological defects, with mitochondria appearing fewer in number and as larger swollen structures. These mitochondria also exhibited reduced membrane potential and a reduction in respiratory chain components. Very similar features were reported to occur in mutants lacking Mdm38p and were attributed to the role that this protein plays in K+/H+ exchange [55] raising the possibility that elevated Tpk3p induces a loss of homeostasis within the organelle.

Our observations suggest that elevated Tpkp3 activity induces a novel mitochondrial state that results in non-respiring mitochondria prone to the production of ROS. A key question arising from this research is therefore, why do mitochondria damaged by elevated Tpk3p activity produce ROS? Interestingly a previous study has proposed that constitutive activation of the Ras pathway, via expression of the *Ras^val19 ^*allele, leads to a state 4 non-phosphorylating respiration that is prone to ROS production [16]. This study also suggested that the respiratory activity and mitochondrial membrane potential of *Ras^val19 ^*cells becomes elevated, offering a plausible explanation as to the origin of ROS [16]. However in actin aggregating cells, which also exhibit constitutive Ras activation, and in *Δpde2 *cells grown in the presence of 4 mM cAMP, there exists little or no mitochondrial membrane potential [27]. This was confirmed by treatment of cells with FCCP, a proton ionophore, that allows protons to flow back through the inner mitochondrial membrane into the matrix. This releases the inhibition caused by the build up of a proton gradient and allows the electron transport system to operate at its maximal rate (ETS). In cells with functional mitochondria, FCCP treatment normally results in an increase in oxygen consumption of around 30-40% (Figure 3A). However cells lacking *PDE2 *failed to respond to FCCP when grown in the presence of exogenous cAMP. This supports our suggestion that elevated Tpk3p activity results in a loss of mitochondrial membrane potential. We would argue that the constitutive activation of Ras/cAMP/PKA signalling can lead to altered mitochondrial function prone to ROS function that does not occur as a result of state 4 respiration.

## Conclusions

In summary, our study has revealed that the loss of regulation of cAMP/PKA signalling leads to cell death that requires *TPK3 *dependent transcriptional remodelling that suppresses stress response signalling and promotes mitochondrial dysfunction. Tpk3p appears to facilitate cell death via action on several downstream targets, notably *HAP4*, which induce the formation of dysfunctional mitochondria which fail to respire and produce high levels of ROS. The loss of respiratory capacity within mitochondria has been associated with a wide range of human disorders including stroke, cardioencephalomyopathy, hepatic failure and Leigh's syndrome [56,57] and is also associated with aging and aging-related degenerative diseases such as Alzheimer's [58]. We hypothesise that the increased production of respiratory incompetent mitochondria that are prone to ROS production may contribute to the pathology of a variety of age related disease states. Further research in this area utilising yeast as a model eukaryote may well provide insights of important medical relevance.

## Abbreviations

(ETS): electron transport system; (TET): Triethyltin bromide; (FCCP): Carbonylcyanide-4-(trifluoromethoxy)-phenylhydrazone; (PKA): Protein Kinase A; (ROS): Reactive Oxygen Species

## Authors' contributions

JEL carried out all of the high resolution respiromtery and ROS measurements included, and in collaboration with CWG made major contributions to every aspect of the paper including microscopy and microarray experiments. Both authors contributed to the conception of the study and to the writing and editing of the manuscript text.

## Supplementary Material

Additional file 1 **Primers used in this study**. Primers used to delete and confirm deletion of the genes *TPK3*, *PDE2 *and *TPK1*. Click here for file

Additional file 2 **TPK3 activity is required to promote loss of respiration**. Respiratory profiles were determined for wild type, Δpde2, Δpde2 Δtpk3 and Δpde2Δtpk3 + *TPK3 *cells grown in the absence and presence of 4 mM cAMP. Values of routine respiration (Routine), respiration facilitated by leak of protons across the inner membrane (LEAK), maximal respiratory rate (ETS), and respiratory control ratio (RCR) are the mean of 3 independent experiments, error bars are S.E. of those means. Values are for oxygen flux (pmols/s/10^6 ^cells). Click here for file

Additional file 3 **Genes down regulated by elevated cAMP/PKA activity**. Complete list of genes down-regulated by 2 fold or more in *Δpde2 *cells but not in *Δpde2 Δtpk3 *when grown in the presence of exogenous 4 mM cAMP for 24 h to diauxic shift. Genes were grouped by GO assignment to a cellular process using the Slim Mapper tool as described in materials and methods. Click here for file

Additional file 4 **Genes up regulated by elevated cAMP/PKA activity**. Complete list of genes up-regulated by 2 fold or more in *Δpde2 *cells but not in *Δpde2 Δtpk3 *when grown in the presence of exogenous 4 mM cAMP for 24 h to diauxic shift. Genes were grouped by GO assignment to a cellular process using the Slim Mapper tool as described in materials and methods. Click here for file

Additional file 5 **Effects of elevated cAMP/PKa activity on Carbohydrate storage genes**. Genes involved in storage carbohydrate synthesis downregulated in *Δpde2 *cells but not in *Δpde2 Δtpk3 *when grown in the presence of exogenous cAMP. Click here for file
