# Supplementary material for: cAMP/PKA signaling balances respiratory activity with mitochondria dependent apoptosis via transcriptional regulation
Source: BMC Cell Biol. 2010 Nov 25;11:92. doi: 10.1186/1471-2121-11-92 (PMC3001716; doi:10.1186/1471-2121-11-92)
Supplement: Additional file 1 — Primers used in this study. Primers used to delete and confirm deletion of the genes TPK3, PDE2 and TPK1. [file 1471-2121-11-92-S1.PDF]

Primer	Sequence
TPK3 deletion 5’	CTTACAAAATGGAAGTGAAGATGTCGCGAACGCTCCGTG GTTTAACGAAGTGATATGG
TPK3 deletion 3’	CCATATCACTTCGTTAAACCACGGAGCGTTCGCGACATCT TCACTTCCATTTTGTAAG
TPK3 deletion check 5’	TGTTTTCCCTGGGTGAAAAG
PDE2 deletion 5’	ATGTCCACCCTTTTTCTGATTGGAATACACGAGATTGAGA AATCTCAAACCAGCTGAAGCTTCGTACGC
PDE2 deletion 3’	CTATTGTGGTTTCTTGTGTTTCATCCAGTATTCTTTATTGAT TTTGAGCATAGGCCACTAGTGGATCTG
PDE2 deletion check 5’	TTGCACTGACGCTTTATTAGCC
PDE2 deletion check 3’	GCACAAATACTTCACAAAAGGAA
TPK1 deletion 5’	GTGCATGAATTATAGCTGATTGTGTGAAAGAATCTTTTTTT TGGGCAGCTGAAGCTTCGTACGC
TPK1 deletion 3’	AAAAATATAGATACGAGAGGAAAATACAACAAAACATTAGT CGCATAGGCCACTAGTGGATCTG
TPK1 deletion check 5’	AGAAGCTGCGATTGTATCCA
Ura3 deletion cassette 3’	TTGGCTAATCATGACCCC
Leu2 deletion cassette3’	ATCTCATGGATGATATCC
