# Supplementary material for: cAMP/PKA signaling balances respiratory activity with mitochondria dependent apoptosis via transcriptional regulation
Source: BMC Cell Biol. 2010 Nov 25;11:92. doi: 10.1186/1471-2121-11-92 (PMC3001716; doi:10.1186/1471-2121-11-92)
Supplement: Additional file 2 — TPK3 activity is required to promote loss of respiration. Respiratory profiles were determined for wild type, Δpde2, Δpde2 Δtpk3 and Δpde2Δtpk3 + TPK3 cells grown in the absence and presence of 4 mM cAMP. Values of routine respiration (Routine), respiration facilitated by leak of protons across the inner membrane (LEAK), maximal respiratory rate (ETS), and respiratory control ratio (RCR) are the mean of 3 independent experiments, error bars are S.E. of those means. Values are for oxygen flux (pmols/s/106 cells). [file 1471-2121-11-92-S2.PDF]

	-cAMP				+cAMP			
	Routine	LEAK	ETS	RCR ETS/LEAK	Routine	LEAK	ETS	RCR ETS/LEAK
WILD TYPE	8.14±0.9	2.17±0.2	12.14±1.6	5.5±0.5	6.06±0.5	1.51±0.1	10.70±0.5	7.1±0.2
Δ pde2	9.77±0.6	2.39±0.1	13.95±1.7	5.8±0.7	1.75±0.6	1.17±0.4	2.15±0.7	2.0 ±0.7
Δ pde2 *Δ* tpk3	7.45±0.5	1.89±0.01	12.04±1.2	6.4 ±0.8	6.22±1.1	2.02±0.3	7.85±1.4	3.98±0.7
Δ pde2 *Δ* tpk3 +*TPK3*	5.60±1.6	1.60±0.7	6.25±1.8	4.3±0.6	0.83±0.7	0.62±0.4	0.72±0.3	1.5±0.6
