# Supplementary material for: cAMP/PKA signaling balances respiratory activity with mitochondria dependent apoptosis via transcriptional regulation
Source: BMC Cell Biol. 2010 Nov 25;11:92. doi: 10.1186/1471-2121-11-92 (PMC3001716; doi:10.1186/1471-2121-11-92)
Supplement: Additional file 3 — Genes down regulated by elevated cAMP/PKA activity. Complete list of genes down-regulated by 2 fold or more in Δpde2 cells but not in Δpde2 Δtpk3 when grown in the presence of exogenous 4 mM cAMP for 24 h to diauxic shift. Genes were grouped by GO assignment to a cellular process using the Slim Mapper tool as described in materials and methods. [file 1471-2121-11-92-S3.PDF]

Cellular Process (Assessed by Gene Ontology)	Genes Down-regulated 2 fold or more
Metabolic process	AAC3, ADH7, ANB1, ATG15, BIO2, BTT1, CPS1, CSN9, CUS1, DAK2, DAL80, ECM11, EDC1, FAL1, GPD2, HEM13, HIS1, HPR1, IRC4, IRC7, LYS9, MET14, MET16, MET17, MET2, MET32, MFT1, MIG2, MIG3, MKC7, NAT5, NDJ1, NRK1, PAU8, PDC5, PHO11, PHO5, PUF3, RBL2, RCK1, RPS22A, SAE2, SAP30, SMP1, SOR2, SPE3, SRN2, TAF3, THI4, THI80, UTR2, YAP6, YEH1, YGK3, YGR287C, YNL108C, ZAP1
transport	AAC3, APL6, BIK1, COG4, COG8, DAL5, DAN1, GIT1, HOT13, HPR1, HXT1, HXT11, HXT2, MFT1, OAC1, RIX7, RUD3, SLO1, RN2, SRP40, YMC2, ZRT1
transcription	DAL80, HPR1, MFT1, MIG2, MIG3, PAU8, SAP30, SMP1, TAF3, YAP6, ZAP1
cell cycle	BFA1, BIK1, BRN1, DAD2, ECM11, HUG1, MAM1, NDJ1, RCK1, SAE2, SCM3, SHE1, SPO22, TEM1, VIK1
cellular amino acid metabolic process	HIS1, LYS9, MET14, MET16, MET17, MET2, MET32, PDC5
signal transduction	BFA1
Not yet annotated	YDL038C
cannot be mapped to a GO slim term	PRM7, SPL2, TIR1, YCR102C
