# Supplementary material for: cAMP/PKA signaling balances respiratory activity with mitochondria dependent apoptosis via transcriptional regulation
Source: BMC Cell Biol. 2010 Nov 25;11:92. doi: 10.1186/1471-2121-11-92 (PMC3001716; doi:10.1186/1471-2121-11-92)
Supplement: Additional file 4 — Genes up regulated by elevated cAMP/PKA activity. Complete list of genes up-regulated by 2 fold or more in Δpde2 cells but not in Δpde2 Δtpk3 when grown in the presence of exogenous 4 mM cAMP for 24 h to diauxic shift. Genes were grouped by GO assignment to a cellular process using the Slim Mapper tool as described in materials and methods. [file 1471-2121-11-92-S4.PDF]

Cellular Process (Assessed by Gene Ontology)	Genes Up-regulated by 2 fold or more
Metabolic process	AAH1, ACS1, ADH2, AGX1, ALD4, ATG4, ATP19, AYR1, CAT8, COX10, COX4, CRC1, CSM4, CYB2, CYT2, DCS2, EDC2, ERF2, FBP1, FLC1, GAC1, GLC3, GND2, GSM1, ICL1, IDP2, IRC15, IZH1, IZH4, KGD1, LSB6, MAM33, MBR1, MCR1, MEF2, MLS1, MRK1, NDE2, OCH1, PCL1, PET10, PIG2, PUT4, REG2, RPI1, RPM2, RPS9A, RTC6, SDH2, SHR5, SIP2, SKN1, SOL4, TAZ1, TPS2, TSL1, USV1, VID28, YAT2, YDR018C, YIG1, YJL045W, YPS1
transport	ADY2, ALY2, ATG4, ATP19, CCC2, ERF2, ESBP6, FLC1, FTR1, HXT5, MCH1, MEP3, NUM1, ODC1, PDR15, PIC2, PMC1, PMP1, PUT4, RSB1, SCO1, SFC1, SHR5, SIT1, SMF1, SMF2, STL1, TPO2, TPO3, WSC4, YOR1
transcription	CAT8, GAC1, REG2, RPM2, USV1, VID28
cell cycle	CSM4, GAC1, IRC15, PCL1, SCM4
cellular amino acid metabolic process	ADH2, AGX1, IDP2, PUT4
signal transduction	GAC1, GPG1, SIP2
biological process unknown	AIM33, BSC2, ECL1, FMP16, FMP33, FMP48, IRC23, JID1, MRH1, OM14, PNS1, RTC2, RTN2, SPG1, STP4, THI74, YDR514C, YER053C-A, YGL010W, YGR067C, YGR079W, YHR033W, YJL160C, YJL181W, YKL096C-B, YKL187C, YKR045C, YLR152C, YLR154W-E, YLR157W-E, YLR164W, YLR297W, YLR307C-A, YMR034C, YMR206W, YNL195C, YNR014W, YOR186W, YPL014W, YPL109C, YPR157W, YRO2, YTP1
Not mapped to a GO slim term	SNQ2, XBP1, YCR061W
