# Supplementary material for: cAMP/PKA signaling balances respiratory activity with mitochondria dependent apoptosis via transcriptional regulation
Source: BMC Cell Biol. 2010 Nov 25;11:92. doi: 10.1186/1471-2121-11-92 (PMC3001716; doi:10.1186/1471-2121-11-92)
Supplement: Additional file 5 — Effects of elevated cAMP/PKa activity on Carbohydrate storage genes. Genes involved in storage carbohydrate synthesis downregulated in Δpde2 cells but not in Δpde2 Δtpk3 when grown in the presence of exogenous cAMP. [file 1471-2121-11-92-S5.PDF]

Process	Gene	Fold Change	Function
Glycogen/trehalose accumulation	GLC3	-1.99	Glycogen branching enzyme, involved in glycogen accumulation;
	PIG2	-2.6	Putative type-1 protein phosphatase targeting subunit that tethers Glc7p type-1 protein phosphatase to Gsy2p glycogen synthase
	MRK1	-3.6	Glycogen synthase kinase 3 (GSK-3) homolog; functions to activate Msn2p-dependent transcription of stress responsive genes and that function in protein degradation
	TPS2	-2.5	Phosphatase subunit of the trehalose-6-phosphate synthase/phosphatase complex, which synthesizes the storage carbohydrate trehalose; expression is induced by stress conditions and repressed by the Ras-cAMP pathway
	TSL1	-2.7	Large subunit of trehalose 6-phosphate synthase (Tps1p)/phosphatase (Tps2p) complex, which converts uridine-5'-diphosphoglucose and glucose 6-phosphate to trehalose, homologous to Tps3p and may share function
